# ‘The Primacy of ‘Home’: An exploration of how older adults’ transition to life in a care home towards the end of the first year

**DOI:** 10.1111/hsc.13232

**Published:** 2020-11-26

**Authors:** Marie O’Neill, Assumpta Ryan, Anne Tracey, Liz Laird

**Affiliations:** ^1^ School of Nursing and Institute of Nursing and Health Research Ulster University Co Londonderry Northern Ireland; ^2^ School of Psychology Ulster University Coleraine, Co Londonderry Northern Ireland

**Keywords:** adaptation, care home, grounded theory, older people, quality of life, transitions

## Abstract

This grounded theory study sought to explore how older adults’ experience the transition from living at home to a care home with a specific focus on the latter part of the first year of the move. The study was carried out within a large Health Trust in the UK between August 2017 and May 2019. Purposive sampling was used in the initial stages of data collection. Thereafter and consistent with grounded theory methodology, theoretical sampling was employed to undertake semi‐structured interviews with 17 individuals from eight care homes between 5 and 12 months after the move. This paper reports five key categories which were: (a) The lasting effect of first Impressions ‘*They helped me make my mind up’* (b) On a Journey ‘*I just take it one day at a time’*, (c) Staying connected and feeling ‘at home’ ‘*You get something good out of it you know…you get hope’*. (d) Managing loss and grief ‘*It was important for me to say cheerio to the house’* and (e) Caring relationships ‘*I didn't realise that I was lonely until I had company’*. Together these five categories formed the basis of the core category ‘The Primacy of ‘Home’ which participants identified as a place they would like to feel valued, nurtured and have a sense of belonging. This study identifies that it is important for individual preferences and expectations to be managed from the outset of the move. Individuals and families need to be supported to have honest and caring conversations to promote acceptance and adaptation to living in a care home while continuing to embrace the heart of ‘home’. Key recommendations from this study include the need to raise awareness of the significance of the ongoing psychological and emotional well‐being needs of older people which should be considered in policy directives and clinical practice.

**What is known about this topic?**

- The extent to which individuals exercise control over the decision to move to a care home is recognised as an important determinant of their relocation experience.
- Most studies report that care home environments can be restrictive, therefore making adjustment and adaptation more challenging.
- There is a dearth of research on the extent to which residents can be facilitated to feel ‘at home’ in a care home environment

**What this paper adds**

- Positive adaptation is connected to older peoples’ perceived quality of life, continued connection to home, family and community, and having opportunities to develop meaningful relationships with staff and other residents.
- Facilitating difficult and caring conversations with individuals and their families is required to manage individual expectations of the move to promote a positive adaptation process.
- Failure to engage early with these difficult conversations can negatively impact on the adaptation process over the course of the first year of life in a care home.
- Older people do not always have existing social supports to cope with bereavement and loss in the care home which has a significant impact on their psychological well‐being.

## 1 INTRODUCTION

The experience of ageing may require older people to make transitions in their living environments, either by adaptations to current homes or through relocations to more supportive environments (Perry et al., 2014). ‘Ageing in place’ relates to an older person’ sense of identity through their independence and autonomy alongside caring relationships and roles in the places they live (Wiles et al., 2012). Older people want choices about where and how they age in place, therefore the importance of involving 'the person' in making the decision to move and having a choice of a care home is significant. Furthermore, having a sense of attachment or connection to their existing home or community maintains security and familiarity (O'Neill et al., 2020a, 2020b; Wiles et al., 2012).

The extent to which individuals, who need 24‐hr care exercise control over the decision to move to a care home is recognised as an important determinant of their relocation experience (Chao et al., 2008; Fraher & Coffey, 2011; Johnson et al., 2010; Lee et al., 2013; O'Neill et al., 2020a, 2020b; Ryan & McKenna, 2015). Individuals have reported that greater involvement in the decision to move to a care home could have eased the negative feelings surrounding the move (Nwe et al., 2011; Sury et al., 2013). Similarly, whether the decision to move was made by the individual or imposed by others adversely affects residents’ transition experience and their related grief reactions to the move and to the loss of their home (Crawford et al., 2015; Pritty et al., 2019; Zizzo et al., 2020).

Most studies report that care home environments can be restrictive, therefore making adjustment and adaptation more challenging for the individual as feelings of institutionalisation can occur (Bradshaw et al., 2012; Cooney, 2012; Ericson‐Lidman et al., 2014; Križaj et al., 2018; Tsai & Tsai, 2008). In contrast to the negative assertions, the literature also suggests that the potential benefits for older people entering into care homes include improved self‐worth, morale, physical functioning, feeling less lonely and feeling more secure (Katz et al., 2011; Lee et al., 2013; O'Neill et al., 2020a, 2020b; Wadensten et al., 2007). A major challenge associated with the transition into a care home is the loss of the individual's home, therefore threatening identity, belonging and sense of self (Brownie et al., 2014; Lee et al., 2013; Westin, 2007). Home is not only fundamental to a person's self‐identity and social relationships, but homely environments are essential to promote recovery, well‐being and quality of life (Böckerman et al., 2012; Molony, 2010; Rioux & Werner, 2011). Additionally, individuals may lose previous social and communication networks (Zamanzadeh et al., 2017) putting them at risk of feeling lonely and isolated (Brownie et al., 2014).

Research undertaken by Cooney (2012) identified four categories as significant to ‘finding home’ in long‐term care settings. These were: ‘continuity’, ‘preserving personal identity’, ‘belonging’ and ‘being active and working’. What made it simpler or more problematic for older people to ‘find home’ was either unique to the individual (adaptive responses, expectations and/or past experiences) or at an institutional level (ethos of care, institutional culture, environment of setting).

Rijnaard et al. (2016) undertook a systematic review of seventeen mainly qualitative research studies. The aim of the review was to provide an overview of factors influencing the ‘sense of home’ of older adults residing in the nursing home. They found that a nursing home resident's ‘sense of home’ was influenced by fifteen factors, divided into three themes: (a) psychological factors (sense of acknowledgement, preservation of one's habits and values, autonomy and control, and coping); (b) social factors and activities (interaction and relationship with staff, residents, family, friends and pets) and (c) the built environment (private space and quasi‐) public space, personal belongings, technology, ‘look and feel’ interior design and the general maintenance, and ‘the outdoors and location’ which relates to the home's outdoor space and the neighbourhood at large. Similar findings were reported in a systematic review by Fitzpatrick and Tzouvara (2019) which used Meleis's Theory of Transition (2010) to explore facilitative and inhibitive influences on older peoples’ transition to long‐term care. Data synthesis of 34 studies identified that the transition featured potential personal and community focused facilitators and inhibitors which were mapped to four themes: ‘resilience of the older person’, ‘interpersonal connections and relationships,’ ‘this is my new home’ and ‘the care facility as an organisation’.

Complex and multidimensional factors can influence the adaption process for older people when relocating to a care home (Bradshaw et al., 2012; Brownie et al., 2014; Križaj et al., 2018; Roy et al., 2018). There is a dearth of research on the extent to which residents can be facilitated to feel ‘at home’ in a care home environment, particularly during the first year of the move. This study sought to address this imbalance.

### 1.1 Aim

To explore how older adults’, experience the transition from life at home to life in a care home with a specific focus on the latter part of the first year of the move.

## 2 METHODS

### 2.1 Study design

A grounded theory approach, consistent with the work of Strauss and Corbin (1990, 1998), was chosen as it facilitated the development of a new perspective on the experiences of older people living in a care home with a particular focus on the latter part of their first year after the move. Grounded theory is recommended when investigating social problems or situations to which people must adapt (Corbin & Strauss, 2008; Maz, 2013; Morse, 2009). Grounded theory is an ideal methodology to understand actions and processes through transitions (Morse, 2009) and has been used by qualitative researchers to study processes engaged in by service users (Grant et al., 2009). A semi‐structured interview schedule was designed to stimulate discussion of individuals’ perceptions, thoughts and feelings about their experiences of living a care home during this specific time period.

### 2.2 Participants and recruitment

The study was carried out within a large Health and Social Care Trust in the UK which provides health and social care services including 1,800 residential and nursing home placements to a population of approximately 300,000 people across rural and urban areas. Purposive sampling was used to recruit participants for the initial interviews. Thereafter and consistent with grounded theory methodology, theoretical sampling was employed to recruit a sample of 17 individuals who had resided in a care home for a time period of between 5 and 12 months. The 5‐month inclusion criterion was important as this was consistent with the time frame for confirmation of permanent residency. Residents who met this criterion were identified by community‐based care managers and through direct contact with care home managers (Table 1).

**TABLE 1:** Characteristics of the Interviewees and details of admission

Pseudonym	Age	Living arrangement prior to move	Individuals account of details surrounding admission to care home	How was admission arranged?
Jane	84	Lived alone in rented accommodation	“Too old to be on my own and I’m frightened of falling”. Jane developed a chest infection was admitted to hospital, and then had poor mobility.	Family arranged admission through G.P/social worker. Did not visit care home prior to admission.
Ellen	82	Lived alone in rented accommodation	Husband died recently. Wanted to move to sheltered housing. Nursing Home was only available choice.	Social worker arranged admission. Ellen wanted sheltered accommodation, but care home was offered as only accommodation available. Did not see care home prior to admission.
David	88	Lived alone in family home.	Chose care home as wife already there a year previously. Health deteriorated after a fall at home “I’m too old to be on my own”	Chose care home for his wife in the first instance then when his health deteriorated he planned the move.
Bernadette	92	Lived alone in family home.	Had fall at home admitted to hospital. “Family thought it was not right for me being on own. Mobility poor‐ “Doctor says move in”.	Daughter's visited the home and recommended it to Bernadette. G.P and social worker decided. Bernadette did not visit home prior to admission.
Andrew	82	Lived alone in family home	Wife died. Had recent stroke. Was taken to hospital. Family overseas.	Daughter came home and visited local care homes. Andrew in hospital prior to admission but did not visit care home.
Martha	80	Lived alone at home	“Fell at home needed a new hip”. Changing family circumstances ‐ no‐one now at home.	Social worker arranged admission. Only care home available did not see care home prior to admission.
Sean	60	Lived with wife and children in family home	Developed sepsis, progressed to paraplegia with lesion on spine. Total nursing care required. Facilities at home do not support nursing care.	Was transferred straight from hospital to care home which was only one available to meet care needs. Did not visit care home prior to admission.
Tracey	88	Lived alone in rented accommodation	Getting worried about deterioration in health or falling, chose residential care admission.	Arranged through social worker who took Tracey to see a few care homes and she chose the one she liked the most.
Molly	80	Lived alone in rented accommodation	“Developed anxiety”. G.P advised admission “feeling safe now”	Was being placed by social worker in a care home a few miles away from her family. Molly waited on a vacancy becoming available locally. Did not visit care home prior to admission.
Charles	83	Lived with wife in rented accommodation	Wife died suddenly who was carer. Had been in a wheelchair for many years due to war injury. Admitted to care home on day of wife's death in a taxi.	Charles was admitted to the care home the night his wife died as she was his carer. It was an emergency admission and he had no say in the move nor did he visit the care home prior to admission.
Anne	90	Lived alone in own home	Admitted to hospital with TIA. Then transferred to nursing Home ‐ “I had no choice”.	Was moved to a nursing home initially post hospital. Anne asked social worker for a transfer to residential care. Did not visit the care home prior to admission.
Isobel	96	Lived alone in rented accommodation	Chest infection admitted to hospital. Reduced mobility in hospital. Son working away.	Isobel stated she would have needed carers at home so social worker asked where she would like to go. Requested care home next to home but no vacancies. Did not visit care home prior to admission.
Therese	78	Lived at home with brother and sister.	Recent stroke. Sister and brother were “too old to care for me at home”.	Therese chose nursing home as she had spent 2 weeks convalescing post‐surgery 3 years previously. Did not visit home prior to admission day.
Tony	87	Lived alone in family home.	Developed pneumonia and was admitted to hospital. G.P advised admission to care home.	G.P and social worker arranged admission. Tony knew of care home because it was local but did not visit the care home prior to admission.
Hugh	83	Lived alone in family home	Accident at home, admitted to hospital. Reduced mobility‐ niece lives far away.	Hugh stated pressure ‘to release hospital bed’ so his Niece visited a few care homes and made arrangements for admission which is 30 miles from, Hugh's home. Did not visit care home prior to admission.
Mona	81	Lived at home with daughter	Poor mobility for many years. Daughter (carer) fell and injured back requiring hospital admission.	Mona was admitted to the care home the day her daughter fell and sustained fractures requiring prolonged hospital admission. Her daughter was her carer. It was an emergency admission and she had no say in the move nor did she visit the care home prior to admission.
Kevin	83	Lived alone in family home	Fell while shopping. Taken to hospital.	Hospital staff advised residential care home admission arranged by social workers. Did not visit care home prior to admission.

### 2.3 Data collection

Detailed information about the research study was presented in writing and verbally to each participant and a binder provided which included information about the study printed in large font with contact details of the research team. Information details included how to lodge a complaint, a consent form and the procedure to be followed in specific situations, for example if the older person became upset or distressed. A tape‐recording of this information was made available to visually impaired residents if required. Individual face‐to‐face interviews were arranged at a time convenient for each participant. All interviews were conducted between August 2017 and May 2019. Semi‐structured interviews were conducted with 17 individuals from eight care homes across the study site. Written consent for each interview was provided by participants. The audio‐taped interviews were transcribed verbatim with each interview lasting approximately 60 min. Field notes were taken by the interviewer. Consistent with a grounded theory approach, the semi‐structured interviews provided both focus and flexibility (Corbin & Strauss, 2008). Simultaneous data collection and constant comparative analysis were repeated until data saturation was accomplished along with the advancement of theoretical concepts. The interview schedule evolved commensurate with category and subcategory dimensions using grounded theory approach (see Appendix 1).

### 2.4 Ethical considerations

Ethical approval for the study was initially obtained through the ethics committee of the university leading the research. Ethical approval was subsequently gained from the regional ethics committee and from the health and social care trust where the study was based. The researcher made concerted efforts through recruitment to ensure that the voices and experiences of older people were given due attention and that participants themselves found their involvement meaningful (Dewing, 2009). Minimal or no cognitive impairment as defined by the Mini Mental State Examination (MMSE > 24) was a criterion for participant's inclusion in the study. The study was carried out over a 12‐month period and relied upon participants ability to recall and reflect upon their experiences over this time frame. By undertaking the MMSE prior to interview the researcher was able to ascertain participant's cognitive ability prior to each interview and any changes over time. Informed consent was provided by each participant with additional consent obtained to use a digital recorder for the interviews. Assurances of confidentiality and anonymity were provided and supported by the allocation of pseudonyms in the presentation of the study and its findings.

### 2.5 Data analysis

NVivo 12 qualitative data analysis programme software (QSR International, 2018) facilitated the organisation, management and retrieval of transcribed interviews and field notes and provided tools for coding, categorising and linking qualitative data. Data analysis was informed by open, axial and selective coding principles as espoused by Corbin and Strauss (2008). Simultaneous data collection and constant comparative analysis were undertaken and repeated until theoretical saturation was achieved when no new categories were identified, and until no new instances of variation for existing categories ceased to emerge. The final stage of selective coding was the process of integrating and refining categories, a core category was identified that related to the other categories, validating those similarities and relationships (Strauss & Corbin, 1998).

### 2.6 Ensuring rigour

The initial interviews were recorded and checked to ensure the rigour of the data collection procedures. The constant comparative analysis of emerging data facilitated the verification of findings and minimised the likelihood of personal bias. The process of theoretical sampling continued until the emerging concepts and categories reached saturation. During the open and axial coding stages, two members of the research team (MON & AT) independently viewed the original uncoded manuscripts and confirmed themes thus ensuring that interpretations represented the experiences of the individuals. After the selective coding process, trustworthiness of the data was enhanced by all the research team who reviewed themes and discussed alternative interpretations of the data to maximise credibility, dependability and confirmability (Lincoln & Guba, 1985). In keeping with one of the tenets of grounded theory (Corbin & Strauss, 2008), individuals’ own language at all levels of coding was used to further ground theory construction and add to the credibility of findings.

### 2.7 Profile of participants

A total of 17 participants in this study comprised of ten females and seven males with an average age of 83.3 years. Seven participants were admitted directly from the hospital. Four of these individuals were female and three were male. In comparison, six female and four male participants were admitted to the care home directly from home (one female and one male moved to the care home when their respective carers became ill). The main reasons cited for prompting the relocation to a care home were deterioration in physical health (*n* = 11), recent bereavement (*n* = 3) and no‐one to take care of me/changing family circumstances (*n* = 3). The majority of the individuals (*n* = 15) did not visit the care home prior to the move; one participant chose the home previously for his wife and one participant was accompanied by a social worker.

## 3 FINDINGS

**FIGURE 1:**
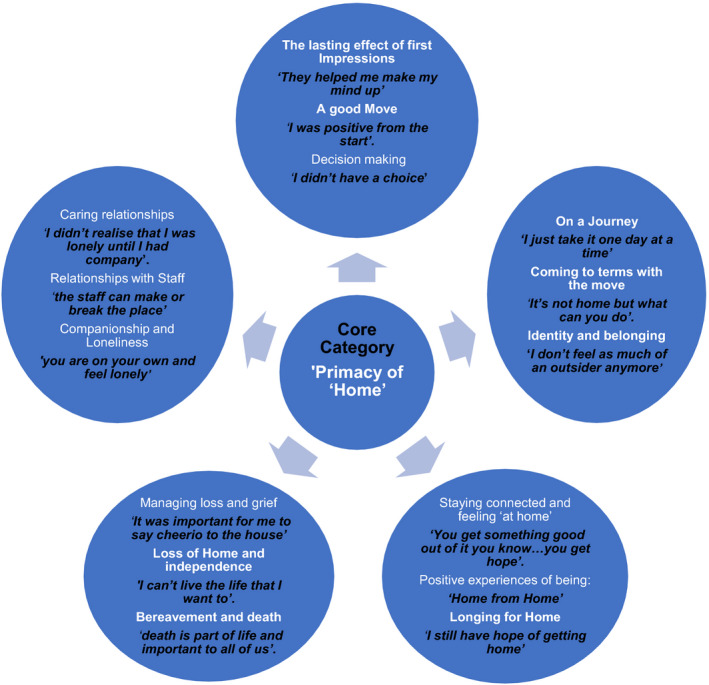
Individuals’ experiences of living in a care home. A diagram illustrating relationship

This paper reports key findings pertaining to the experiences of older people between 5 months and 12 months after the move to a care home. Identified categories were as follows: (a) The lasting effect of first Impressions: ‘*They helped me make my mind up’* (b) On a Journey: ‘*I just take it one day at a time’*, (c) Staying connected and feeling ‘at home’*:* ‘*You get something good out of it you know…you get hope’*. (d) Managing loss and grief: ‘*It was important for me to say cheerio to the house’ and* (e) Caring relationships: *‘I didn't realise that I was lonely until I had company’*. Together these five categories formed the basis of the core category the ***‘The Primacy of ‘Home’*** which encapsulates the experiences of the participants who placed significance on maintaining their identity and having a sense of belonging both within the care home and with their continued connections with ‘family and home’ (Figure 1).

### 3.1 The lasting effect of first Impressions – ‘*They helped me make my mind up’*

A good Move‐ *‘I was positive from the start’*.

First impressions of the move to the care home, and the home itself, were important and continued to influence participants’ experiences throughout the first year of the move. Many expressed positive feelings about living in the care home from the outset of the move. This optimism was fostered by the participant and by hospital and care home staff who promoted and endorsed the positivity of the move for the individual concerned.

“I have always loved it here. Ever since I was told by the staff in the Hospital that I was coming here…. they told me that I would love it and I did…… Yes, and they helped me make my mind up about moving here too. Because I was struggling you know on my own and they helped me realise how hard it was trying to carry on. Aye definitely, I was positive from the start” (Kevin, 5 months).

“I remember that from the very beginning the staff were welcoming, they were kind and it just felt right to be here. I know it was my decision to come here but I made a good choice, and I was determined to get on well” (Martha, 12 months).

“Well I suppose I always thought that it was like home from home here. I feel happy here, everyone is friendly, and they are good to all the people here” (Isobel, 11 months)

Decision‐making‐ *‘I didn't have a choice’*.

Up to 12 months after the move to the care home, some participants still maintained that a key factor in their feeling ‘at home’ was whether they were involved in the initial decision to move into the facility. Participants’ comments suggested that the decision was sometimes a forced choice. Exclusion from the decision‐making process and having an overall negative first impression appeared to permeate their experience of the move over the first year.

“Well I suppose people would need to want to come here. I didn't have a choice. It would probably be easier if you had to time to get used to it. But sure, I suppose in the end it's the same outcome …. it's just that it would have a been a smoother journey for me of getting here, if I had had a say in everything. No matter what, well…. you just have to give into it all in the end” (Charles, 12 months).

“Well, I want me and my daughter to be together. I will not settle until then, until she comes here, or I get home. Everybody is still making the decisions for me. I didn't want to come here. I feel lost and nobody cares that I am ill, I feel that this is the end” (Mona, 11 months).

### 3.2 On a Journey—*‘I just take it one day at a time’*

Coming to terms with the move ‘*It's not home but what can you do’*.

It was evident during interviews that some participants were resigned to the fact that there was no other option for them but to be living in the care home. In some cases, they appeared to ‘have come to terms with the move’ voicing acceptance, resignation and ultimately contentment.

“I mean I would want to be at home, but I can't, and this is a good place. Sure, my family can visit me, and they can come and go whenever they want to see me so, what more do I want? …. Sure, what can you do, you just have to get on with it. I will be ninety‐three now in October. I am happy” (Bernadette, 11 months).

“I am probably getting more used to it now. It's not home but what can you do. I suppose I have to reconcile myself with being here now, it's better than being on my own so I have come to terms with living here now……you just have to get on with it and try to make it work” (Hugh, 5 months).

“I just adapted myself to the place and the people. It's hard without my wife but I had to do it. Once you have done six months service here you try to kind of accept it. You get used to it I suppose. I think that it's alright living here now, it's a necessity and I get on with it, I am doing ok” (Charles, 12 months).

For all participants getting settled into ‘their new home’ was a process that occurred over time and individual responses varied. There was no set time for familiarisation to begin and end.

“I think I have settled in well now; it took a while to get used to it all, the people you know and their way of doing things, but I think that I am doing ok now” (Anne, 5 months)

“I love it here now; I think that I have settled in well. It is the best place for me. I couldn't be on my own. I realise that now” (Molly,6 months)

For other participants, their own a personal level of resilience and positive thinking promoted positive adaptation and acceptance experiences to living in the care home.

“I think that you have got to concentrate on the positive things and try not to dwell on the negative things like a bad carer…they are not perfect” (Andrew, 12 months)

“I am making the best of it sure what can you do. I have my things here that's all I want, and I will be seeing out my time here, so I might as well get on with it. You have to be resilient yourself to cope with being in a care home and being on your own” (Tony, 11 months).

Identity and belonging ‘I don't feel as much of an outsider anymore’.

Participants identified the significance of feeling a sense of identity and belonging within the care home and the importance of getting on with residents and care home staff.

“I don't feel as much of an outsider anymore. I am one of the longer stay residents here now. You feel for the new people coming in that they are not yet part of the home” (Tracey, 11 months)

“I would say that you need to try and get involved with the staff and the other people here to help you. You need to talk to the staff an let them know what you want. It is about people making you feel welcome and that you belong here” (Charles, 5 months).

### 3.3 Staying connected and feeling ‘at home’ —*‘You get something good out of it you know…you get hope’*

Twelve months after the move participants spoke about the importance of maintaining connection to their own home, family, friends and community which enabled them to move towards a positive adaptation and acceptance of living in the care home. Participants reported that they were dependant on family or friends to pursue home visits or activities outside of the home. The impact of getting a visit home on participants psychological well‐being was highlighted in many of the interviews. Some participants expressed that the excitement of ‘getting home’ and having respite from the care home, helped in breaking the routine of daily care home life.

“I go out to my daughter's place and home too. The neighbours are there as well you see. Mrs D**** and all them ones you know. So nice to see them and you feel great when you come back” (Bernadette, 11 months)

“My son takes me out to the town or back to the house for a while. I love getting home and I see the neighbours and friends. I always look forward to getting a run out in the car” (Tracey, 11 months)

“I think that people should be taken out of this house even for a while. I get home when my children come over to visit. You get something good out of it you know…you get hope” (Andrew, 5 months)

Positive experiences of being *‘Home from Home’*.

Others reported satisfaction with the care they received in the care home and related this to their ‘own home’ comparing their experiences as being in ‘a home from home’:

“Well I suppose I always thought that this was like home from home when I came here. I feel happy here, everyone is friendly, and they are good to all the people here” (Isobel, 11 months)

“I feel just great…. as I say I just try to keep going. I am ninety‐one and a half now so if god gives me life then I will keep going. And sure, it's like being in my own wee home here, with the care I get here which is so good, I am bound to keep going” (Anne, 12 months).

Longing for Home *‘I still have hope of getting home’*.

Despite the permanent nature of the placement, two participants yearned to go back home at some point in the future. Sean aged 60 years was admitted to the care facility from the hospital following a spinal injury. He had complex health needs and was unable to be cared for at home by his wife who lives alone. Sean wanted to go home if a care package was able to be put in place. In the 12 months he was resident in the care home, he returned home for several days at key holiday periods, that is Christmas, Easter and family occasions. The care home staff provided home care to Sean in the absence of Trust community care provision being available to ensure that Sean got home to his family. A care package for Sean to remain at home permanently has not materialised since.

“I could never resign myself to being here for life. Hope of getting home is fading fast. The best I can think of right now is to get out for weekends. Getting home permanently, well that's the dream. I am losing hope of ever getting a care package organised to be honest. One of the impressions I get from them is that they don't consider my going home to be an option” (Sean, 12 months).

David aged 88 years lived alone and was admitted to hospital following a fall at home. He still had hopes of returning home 5 months after moving to the care home, if his health becomes conducive to independent living. David stated that he could live independently if house adaptations were undertaken including servicing a stair lift. This longing for home remained, even though David had chosen the care home for himself and his wife (who moved in 1 year prior to David and remained a resident in the care home). At 5 months David had expressed dissatisfaction with perceived restrictive care practices that were influencing his independence and autonomy.

“I still have hope of getting home, and I think if they would do something about my hands I would be away and could manage on my own with a bit of home help and then worry about everything else after I get home. I think that there are too many rules and regulations here, your life is not your own and you are not allowed to move about without having to ask for permission” (David, 5 months).

However, at the 12‐month interview, David stated that he ‘could mobilise independently’ and restrictions were lifted, that is he was allowed out to garden on his own and could now mobilise independently within the care home environment. He became more content to live in the care home stating:

“I have got my independence back again. The sanctions have been lifted. I don't think that I will ever be home again now. It has come naturally to me…… It just takes time to come to that realisation……Things are better now” (David, 12 months).

### 3.4 Managing loss and grief *‘It was important for me to say cheerio to the house’*

Loss of Home and independence ‘*I can't live the life that I want to’*.

Saying goodbye to home was very important for some participants. Kevin who had moved to the care home from hospital following a fall was enabled by the social worker to visit his own home on several occasions to sort out his belongings and then a solicitor facilitated his house sale.

“It was good to go down home and get my things together. I brought everything that I wanted up here. It was important for me to say cheerio to the house, I had lived there for 34 years” (Kevin, 5 months)

For one participant Ellen, the perceived loss of her ‘independence and life’ influenced her choice of staying in the care home. Ellen had originally wanted to move into sheltered housing but there were no vacancies available before or during her 12 months stay. Nine months after moving in, Ellen made plans to go to Australia to live with her daughter.

“I am not happy here. I have no independence and I can't live the life that I want to. I can't live here for the rest of my life. I have to have a life. Lots of people live here, they haven't got a life, they're here to stay. I'm not ready for that yet” (Ellen, 11 months).

Bereavement and death ***‘*** *death is part of life and important to all of us’*.

The impact of the first year of life in a care home was, for some participants, compounded by the death of close family members before and after the move. For healthcare practitioners, death is an inevitable part of working in a care home, but this study suggests that the grief and loss experienced by residents after the death of fellow residents is not always understood by staff.

“There is a change of atmosphere here when someone dies. You miss some more than others, one man called Max he was outgoing, he didn't mind telling you what he thought…. (laughs), we really missed him when he went (Charles, 5 months).

Participants reported experiencing a range of emotions when someone died within the home. They reported feeling ‘upset and annoyed’ when a member of their ‘care home family’ passed. Participants related that staff don't always acknowledge when someone dies and consequently may not provide much‐needed support to residents.

“When people die here in the home the staff don't really talk too much about it…. No, they don't talk about death here at all you know. I suppose everybody is scared of death, we all are, and they don't really want to hear about it. But death is part of life and important to all of us (Ellen, 5 months).

I get especially emotional about people dying in here, like when I see that there's two coffins that have gone past my door. Then I wait for them to come back down again you know…. I get upset when they are taking them to the hospital or wherever they take them… and you see, you start to get used to these things…. going back and forward. I can see that out of the door (Bernadette, 5 months).

### 3.5 Caring relationships: *‘I didn't realise that I was lonely until I had company’*

Maintaining existing relationships and building new ones*‐ ‘I like having the company’*.

Relationships with family and friends were imperative in determining the quality of life for participants. These relationships enhanced their continued emotional well‐being and sense of fulfilment within the care home.

“Well my family are good to me; they do anything I ask and take me out home to visit and come in here too. But sure, I was living on my own so that wasn't good. I have my family and that at the end of the day that is what is important to me” (Tony, 5 months).

“My son never left my side at the beginning; he was always popping in to make sure that I was alright and that I didn't feel lonely here. He still does… I think that he doesn't want me to feel that I am abandoned, and you know what, that really helped me settle” (Tracey, 5 months).

Participants articulated that fellow residents had the potential to influence their lives positively, encouraging a sense of well‐being for themselves and amongst the ‘care home family’ in general.

“I am friends with everyone in here. I like having the company. There are a couple of old ladies, well four or five of us that get together in the wee sitting room and chat away. I like that. It's the new sitting room. We go up there on a Tuesday and sit and chat and play music you know” (Anne, 12 months).

“I am friendly with George next door; we don't do much together ……. But well we go down to the dining room to have dinner and I always talk to him then. I look forward to that” (Charles, 11 months)

“I am getting on great with all the residents and staff. I love it here. They are like a family here; everyone here makes you feel as if you are part of a big family” (Kevin, 5 months)

Relationships with Staff ‐ ‘*the staff can make or break the place’*.

Participants also recognised the significance of supportive and respectful staff who ‘made them feel at home’ from the outset of the move and on a day‐to‐day basis.

“I think the staff are very important. They helped me to feel at home from the beginning. I can tell you, every single day since I have been here, they have been more than good to me” (Anne, 5 months)

“Anything you need, well you just go down to the office and they will help you. They also come and remind you what things are going on, so you don't miss out. I would go so far as to say that it is perfect. It's a long time since I was this happy” (Isobel, 12 months).

“The staff treat me, what's the word? with respect. Yeah. There's more people that have had bad treatment in care homes but not me” (Kevin, 12 months)

“Well you can't fault the staff. I mean they're brilliant. They're all very good to me and they have a great sense of humour……and they always take time to talk to me” (Sean, 5 months)

Positive relationships between staff and residents were identified, with most participants describing staff as caring, kind and influential in making them ‘feel at home’. However, some participants also perceived that some care staff were authoritative and uncaring. Charles, who is from a military background, had his own unique way of expressing his relationship with staff.

“Some of the staff are like the Gestapo, I wouldn't cross them at all. Others are just great. They would go out of their way to help you and make sure that you are alright, but they are so busy and really, they don't have much time. The manager is very good. You have got to have confidence in the people who care for you. That is important. They need to know what they are doing. I have always said that the staff can make or break a place and that's true” (Charles, 5 months).

“Relationships with staff are very important. You either like them or hate them that's the way. There is only one or two of them that you would say to yourself ‘I hope I don't get her today’ because they don't care about you at all, and that's just the way it is (Laughs). I have to say though that most of the staff are pretty good. They are the most important people” (David, 12 months).

Participants highlighted the negative emotional impact of losing ‘favourite’ or ‘good’ staff when they moved on to other employment. Some participants also spoke about the difficulty in developing relationships with staff due to a high turnover of staff in the care home.

“The staff definitely make it more homely. Do you know John do you? he is one of the nurses here, Och he's a great fella and gets everybody going. He's just lovely, but he is leaving now, he got another job in a different care home. That's the problem staff are changing all the time, you don't really get to know them as they don't stay that long” (Martha, 12 months).

“Usually the staff are quite good here, but I think they're short of staff. Two left at the weekend… they're going to work in the hospital. I'm wondering if they are paying them better elsewhere, simple as that…….The odd time they will tell you if they are going ….you feel for them… the last chap that left, he was very well liked. We'll miss him because he was very good” (Therese, 12 months).

Companionship and Loneliness ‘*you are on your own and feel lonely’*.

Many participants had experienced loneliness prior to moving to the care home and new friendships within the home significantly contributed to their sense of home, quality of life and well‐being.

“Life is good now. I have accepted that I am staying here for good. I am happy…. I will not be going anywhere. You know … I didn't realise that I was lonely until I had company” (Kevin, 12 months)

However, some participants still experienced loneliness, in particular those who had no family of their own, or individuals who had no shared interests with other residents.

“I think what makes a big difference when you're living in a care home is if you don't have any family, you are on your own and feel lonely. I mean you can see here at the weekend that nearly everybody gets visitors you know, and I get nobody because I have no family here” (Ellen, 5 months).

Many participants spoke about getting to know other residents’ families and friends when they came to visit and how this enriched their lives also. These interactions contributed to their appreciation of a ‘care home family’. One participant recounted losing ‘care home family friends’ after their relative had died which upset her emotional well‐being leading to isolation and loneliness.

“A man I knew whose wife was upstairs died there recently; it was very sad. The family used to come in to see me too every day at dinner time. I miss them coming in, as I feel lonely some days. I don't really have anyone here to talk to” (Bernadette, 11 months).

## 4 DISCUSSION

This study set out to explore the experiences of 17 older adults following transition from home to living in a care home with a specific focus on the latter part of the first year of the move. Given the paucity of research concerning the adaption process for older people moving to a care home (Bradshaw et al., 2012; Brownie et al., 2014; Križaj et al., 2018; Roy et al., 2018), the aim was to discover more about the extent to which older people can be facilitated to feel ‘at home’ in a care home environment. Five distinct categories captured the experiences between 5 and 12 months after the move to a care home. Identified categories were as follows: (a) The lasting effect of first Impressions—‘*They helped me make my mind up’* (b) On a Journey—‘*I just take it one day at a time’*, (c) Staying connected and feeling ‘at home’—‘*You get something good out of it you know…you get hope’*. (d) Managing loss and grief ‘*It was important for me to say cheerio to the house’* and (e) Caring relationships ‘*I didn't realise that I was lonely until I had company’*. Together these five categories formed the basis of the core category ‘The Primacy of ‘Home’ which encapsulates the experiences of the participants who placed significance on having a sense of belonging both within the care home and with their continued connections with ‘family and home’. These findings concur with international research that has identified how the loss of an individual's home, can compromise identity, belonging, sense of self (Brownie et al., 2014; Lee et al., 2013; Österlind et al. 2017), well‐being and quality of life (Böckerman et al., 2012; Molony, 2010; Rioux & Werner, 2011). Additionally, the loss of an individuals’ previous social and communication networks (Zamanzadeh et al., 2017) can put older people at risk of feeling lonely and isolated (Brownie et al., 2014).

### 4.1 The primacy of home

Establishing a sense of belonging or ‘finding home’ in a care home involves a process of adjustment (Cooney, 2012; Lindley & Wallace, 2015) and has a significant psychological and emotional impact for the individual concerned (Cooney, 2012; Falk et al., 2013; Marshall & Mackenzie, 2008). Moreover, the aptitude to feel at home in care home settings is said to influence residents’ perceived quality of life (Bowers et al., 2009; Hedayati & Khazaei, 2014; James et al., 2014; Tester et al., 2004). The concept of ‘home’ is complex, and has been explored from gerontological, environmental and psychological viewpoints (Moe et al., 2013). Home is not simply a physical space, but it also denotes a meaningful ‘place’ which embodies physical, personal and social dimensions (Wahl & Oswald, 2010); extending beyond the household itself to encompass the neighbourhood and wider community (Bigonnesse et al., 2014). Additionally, Lovatt (2018), found that rather than the meaning of home being inherent in objects, or felt subjectively by residents, meaning is generated through ongoing, everyday interactions between the two. She suggests ‘that life goes on’, and that residents continue to ‘do home’ by actively turning the spaces of their rooms into places of home through habitual practices and by adding to their material surroundings albeit within a different setting and with more limited capabilities.

Participants spoke about the importance of maintaining the connection to their own home, family, friends and community which enabled them to move towards a positive adaptation and acceptance of living in the care home. Even 12 months after the move to the care home, participants identified the positive effect on psychological well‐being of getting a visit home and having respite visits from the care home. This is significant as often the focus of family and care home staff is on trying to ‘replace’ home by creating a ‘home from home’ environment rather than embracing the importance of an older person maintaining their connections to home, family and community which enhances their continued emotional well‐being and sense of fulfilment within the care home. Within this study, 11 out of the seventeen participants visited their own home or a relatives’ home on a regular basis (1–3 monthly) and three participants undertook activities in the community on a weekly basis. All these participant visits were instigated, planned and participants accompanied by family and friends.

'These findings generally resonate with research undertaken by Cooney (2012) who identified the determinants for ‘finding home’ as being ‘continuity’, ‘preserving personal identity’, ‘belonging’ and ‘being active and working. Cooney informs that the potential to ‘find home’ is affected by mediating and facilitating/constraining factors. In addition, Rijnaard et al., (2016) found that a sense of home is influenced by psychological factors including preservation of one's habits and values, autonomy and control, coping and social factors, interaction and relationship with staff, residents, family and friends. The importance of maintaining the connection to home, family and community has also been recognised within Paddock et al. (2019) study when they explored how life in a care home can affect the identity of care homes residents. They found that most residents had little contact with anyone outside of the care home and thus were unable to maintain identity‐affirming connections. They advocated that care homes have the potential to accommodate a multitude of identities by facilitating links with previous social networks or symbols that are necessary to maintain a sense of self within the care home.

In this study the’The Primacy of ‘Home’ is central to residents’ positive adaptation to living in a care home, this involves having the right to make choices about their lives and to take risks. Respecting people's basic human rights to dignity, freedom and respect underpin good quality health and social care. The World Health Organisation (WHO) advocate that healthcare systems should be systematised around older people's preferences and needs, designed to enhance older peoples’ intrinsic capacity and integrated across settings and care providers (WHO, 2015). Moreover, the National Institute for Health and Care Excellence (NICE) (2015) advocate that an individual's care plan should include ordinary activities outside the care home to encourage participation in the community, reduce social isolation and build personal confidence and emotional resilience. This may, however, present challenges for healthcare professionals who have a responsibility to ensure residents’ safety and may influence their decision‐making when accommodating clients’ requests for outdoor activity (Mapes, 2017). Furthermore, it is recognised that despite risk being part of healthcare, nurses may be opposed to taking risks with innovative approaches to care provision due to public or professional criticism should things go wrong (RCN, 2013). It is also recognised, however, that residents are normally dependent on family and friends to support their engagement with activities outside the care home and without this input they may not leave the care home (Paddock et al., 2019). In essence, care home staff usually do not have the staffing levels to make community connections a reality for residents. Furthermore, care homes are highly regulated environments and nursing, and healthcare staff must follow policy directives, guidelines and recommendations for best practice. That said, there is a need to balance people's human right to make choices by facilitating the needs and preferences of older persons and involving them in decision‐making. Commissioning bodies have capacity to influence the way care services are organised and delivered and can stipulate specific practice and outcomes aimed at protecting and promoting human rights.

### 4.2 A sense of belonging

For all participants getting settled into ‘their new home’ was a process that occurred over time and individual responses varied with no set time for familiarisation to begin and end. For some older people, a sense of belonging in their ‘new home’ was experienced from the outset, and these positive first impressions continued to influence their experiences throughout the first year of the move. Many participants within this study spoke about how their own a personal level of resilience and positive thinking promoted positive adaptation and acceptance experiences to living in the care home. A key factor in participants feeling ‘at home’ was whether they were involved in the initial decision to move and choice the facility which appeared to permeate their experience of the move thereafter. This finding is also firmly endorsed within the literature (Chao et al., 2008; Cooney, 2012; Fraher & Coffey, 2011; Johnson & Bibbo, 2014; Johnson et al., 2010; Lee et al., 2013; O'Neill et al., 2020a, 2020b; Ryan & McKenna, 2015; Tanner et al., 2015).

It has been identified that key indicators of residents’ acceptance and adjustment to the care home include the ability to establish a sense of home, maintain self‐identity and self‐worth and develop positive relationships with peers and staff (Cooney, 2012; Falk et al., 2013; Graneheim et al., 2014; Križaj et al., 2018; Molony, 2010; Mortenson et al., 2016; Roberts & Bowers, 2015; Shin & Hyun, 2015). Within this study, participants identified the importance of having good relationships with staff and residents with most participants describing staff as caring, kind and influential in making them ‘feel at home’. However, some participants also perceived that some care staff were authoritative and uncaring. One participant (Charles) stated, ‘the staff can make or break the place’. Furthermore, participants articulated that fellow residents and their families and friends had the potential to influence their lives positively, encouraging a sense of well‐being for themselves making them feel at home amongst the ‘care home family’. These attributes are reflected within Nolan et al.’s (2006) Senses Framework when considering how positive relationships can be created and sustained. They put forward that in order to have a'sense of belonging’, older people need to have ‘opportunities to maintain and/or form meaningful and reciprocal relationships, to feel part of a community or group as desired’.

### 4.3 Making a good move

Some participants in this study expressed positive feelings about living in the care home from the outset of the move. This optimism was fostered by the participant themselves and by hospital and care home staff who promoted and endorsed the positivity of the move for the individual concerned. For other participants, their own a personal level of resilience and positive thinking promoted positive adaptation and acceptance experiences to living in the care home over time. However, it is clear that for some participants in this study the move was not of their choosing, had not turned out the way that they had hoped, or felt ‘cheated’ that they had not been placed in the type of home or locality they wished to be. These core issues prompted one participant (Ellen) to make plans to leave for Australia and another (Sean) to spend his days longing for home and waiting on a care package that did not materialise, perpetuating his unhappiness. It is evident from the literature that a more successful transition or adjustment to a care home is associated with a planned admission rather than unplanned admission, (Gilbert et al., 2015; Koppitz et al., 2017; Walker & McNamara, 2013). Furthermore, it has been positively endorsed within the literature that a person will perceive their relocation more positively after being introduced to a care home prior to the move (Graneheim et al., 2014; Sury et al., 2013; Sussman & Dupuis, 2014). There is an important message to be said about collaborating with individuals and their families to identify individual expectations post move to the care home. Some perhaps difficult and caring conversations need to take place prior to and during the move about future care needs, as not identifying or disregarding people's views and opinions can create a poor adaptation (Bradshaw et al., 2012; Brownie et al., 2014; Križaj et al., 2018).

### 4.4 Psychological and emotional well‐being

Within this study, ‘The Primacy of ‘Home’ is identified as the importance of residents making and maintaining 'connections' thus promoting their mental health and well‐being. Core to understanding how a positive adaptation can be made is recognising the significance of the need for a continuing relationship and continuing connection with home, family and community. Residents need and want something to look forward to, either in terms of getting out for a while with family; or family coming in to visit and spend time; and the opportunity to form relationships with other residents and care workers. It is recognised that loneliness and isolation are key issues associated with admission to long‐term care (Brownie et al., 2014; Cooney, 2012; Hanratty et al., 2018; Križaj et al., 2018; Sury et al., 2013). Conversely, many participants in this study experienced loneliness prior to moving to the care home and friendships developed thereafter significantly contributed to their ‘sense of home’, quality of life and emotional well‐being. However, some participants still experienced loneliness up to a year after the move, particularly those who had no family of their own, or individuals who had no shared interests with other residents. Moreover, those participants who felt that they ‘had no choice’ about the move or choice of care home were still reporting feelings of sadness, regret and lowered mood (Bowers et al., 2015; Brownie et al., 2014; Thein et al., 2011). Grief is a normal process of reacting to a physical loss, such as a death, or a social loss including a relationship. Bereavement is the period after a loss during which grief and mourning occur. The time spent in bereavement for the loss of a loved one depends on the circumstances of the loss and the level of attachment to the person who died (Cassarett et al., 2001). It is noted that a higher prevalence of complicated grief is indicated in the age range of 75–85 years (Newson et al., 2011), denoting more difficulty coping with a loss. Research indicates that older people go through multiple losses of family and friends within their lifetime (Shear et al., 2013) with the transition to widowhood considered the most stressful adjustment to make in later life (Silverstein & Giarrusso, 2010). Moreover, many older people experience bereavement, loss of home and loss of physical function when they move to a care home and also when other residents die within the home (Reed et al., 2002). Within this study, some participants were also grieving loss of family members and others were trying to cope with loss associated with ageing, loss of home and loss of independence. These findings resonate with literature that suggests numerous losses including bereavement are common in old age and present emotional, physical and practical challenges (Ebrahimi et al., 2015; Nicholson et al., 2012; Shear et al., 2013; van Humbeeck et al., 2016). Some participants identified how they were positively supported by social workers and care home staff to control how and when they said goodbye to their house when they were ‘letting go of home’ and how this enabled a successful adaptation to their ‘new home’. In contrast, not all participants had existing social supports to cope with bereavement and loss in the care home. This study suggests that the grief and loss experienced by participants when family, friends or a member of ‘care home family’ dies is not always understood by staff.

The findings from this study clearly identify that older people's experiences of care transition vary, in terms of their support needs and their adaptation to the care home. Our study has identified that individuals’ perceptions of ‘The Primacy of ‘Home’ is connected to their perceived quality of life, continued connection to home, family and community, having opportunities to develop meaningful relationships promoting a sense of belonging amongst their ‘care home family’. The aptitude to feel at home includes control, autonomy and supportive staff relationships and is endorsed within the literature, (Bowers et al., 2009; Bradshaw et al., 2012; Brandburg et al., 2013; Cooney, 2012; Ericson‐Lidman et al., 2014; James et al., 2014; Krizaj et al., 2018). This study is significant because the data were collected up to a year after moving to the care home and there is limited research that has explored the experiences and perspectives of older people up to this time period.

## 5 CONCLUSIONS

In this study ‘The Primacy of ‘Home’ is identified as a place where participants feel valued, nurtured and have a sense of belonging. Older people living in care homes should not be seen as a homogeneous group with a single set of requirements. Moreover, it is very important that older people are enabled to realise their individuality, ability and potential, should that be within the care home or by having continued connection to family and community. Managing individual preferences and expectations from the outset can enable older people to move towards acceptance rather than disabling them which leads to disillusionment and unhappiness. First impressions are very important and have been shown within this study to influence participants’ experiences throughout the first year of the move leading to a more positive adaptation journey. Key recommendations from this study include the need to raise awareness of the significance of the ongoing psychological and emotional well‐being needs of older people which should be considered in policy directives and clinical practice. This could include identifying initial and ongoing expectations for each resident as opposed to service provision facilitated by a senior member of the care home staff with overall responsibility for working in partnership with individuals and families. A designated person should be identified to support and facilitate the psychological and emotional needs of the older person going through bereavement and loss. In addition, individuals and families need to be facilitated and supported to have continued honest and caring conversations to promote acceptance and adaptation to living in a care home while continuing to embrace the heart of home. Initiatives such as My Home Life, an international programme that aims to promote quality of life and positive change in care homes, offers leadership support to provide managers with the knowledge and skills to inspire and lead culture change in care homes (Penney & Ryan, 2018). My Home Life (www.myhomelife.co.uk) advocates that ‘best practice together’ can be developed by focusing on relationships and having meaningful dialogue and interaction with older people and families. The Caring Conversations Framework (Dewar & Nolan, 2013) enhances this engagement and interaction. The framework suggests that, in order to deliver compassionate and dignified care, people need to Be Courageous (giving things a go), Celebrate (finding what worked well), Connect Emotionally (finding out how it made the person feel), Be Curious (understanding what is happening), Collaborate (working together), Consider Other Perspectives (what do others think?), and Compromise (what is real and possible?). It also supports a different attitude to risk‐taking and devising new approaches to problems. In everyday practice, providing quality care relies on care home staff applying their values, attitudes, knowledge and skills. Care home managers are central to this process and with support, can bring about the culture change required to create the sort of ‘home like’ environment highlighted by participants in this study.

## CONFLICT OF INTEREST

The author(s) declared no potential conflicts of interest with respect to the research, authorship, and/or publication of this article.

## AUTHOR CONTRIBUTIONS

All authors have agreed on the final version and meet all four criteria for authorship of the following criteria (ICMJE [http://www.icmje.org/recommend ations/browse/roles‐and‐responsibilities/defining‐the‐role‐of‐authorsand‐contributors.html]): Substantial contributions to conception and design, acquisition of data, or analysis and interpretation of data. Drafting the article or revising it critically for important intellectual content.

## Data Availability

The data that support the findings of this study are available on request from the corresponding author. The data are not publicly available due to privacy or ethical restrictions.

## Appendix 1. Interview schedule

Prompts were used to generate discussion and included:

- Tell me about your experiences since coming to live in the care home?
- To what extent do you feel involved in making decisions regarding your care?
- Whom and what things do you value?
- How do you feel about living in the care home now?
- What helped you adjust to life in the care home?
- What are your plans for the future?
- Prompts on physical, psychological and social well‐being since last interview.
